# Long–term effects of gastric bypass and sleeve gastrectomy in type 2 diabetes: a matched retrospective cohort study from Sweden

**DOI:** 10.1016/j.lanepe.2025.101430

**Published:** 2025-08-30

**Authors:** Ala Mejaddam, Hanne K. Carlsen, Ingrid Larsson, Katarina Eeg-Olofsson, Moa Lugner, Johan Ottosson, Erik Stenberg, Gudrun Höskuldsdóttir, Björn Eliasson

**Affiliations:** a Department of Internal Medicine and Clinical Nutrition, Institute of Medicine, Sahlgrenska Academy, University of Gothenburg, Sweden; b Department of Medicine, Sahlgrenska University Hospital/Östra Hospital, Gothenburg, Sweden; c Centre of Registers Västra Götaland, Gothenburg, Sweden; d Department of Medicine, Sahlgrenska University Hospital, Gothenburg, Sweden; e Department of Surgery, Faculty of Medicine and Health, Örebro University, Örebro, Sweden

**Keywords:** Obesity, Overweight, Weight-loss, Weight-loss treatment, Bariatric surgery, Roux-en-Y gastric bypass, Gastric bypass, Sleeve gastrectomy, Diabetes mellitus, Type 2 diabetes mellitus, Post-operative complications, Long-term complications, All-cause mortality, Overall mortality, Cardiovascular death, Cardiovascular disease, Heart failure, Ischaemic heart disease, Chronic kidney disease, Cancer, Malabsorption, Micronutrient deficiency, Psychiatric disorders, Depression, Anxiety, Alcohol use disorder, Drug use disorder, Gastrointestinal reflux disease, Gastrointestinal ulceration, Bowel obstruction, Wound complications, Gastrointestinal leakage

## Abstract

**Background:**

Long-term data on the efficacy and safety of Roux-en-Y gastric bypass (RYGB) or sleeve gastrectomy (SG) in people with type 2 diabetes mellitus (T2DM) are still limited. Using a matched cohort design, we aimed to evaluate the long-term effects of RYGB and SG on individuals with T2DM, focussing on obesity- and surgery-related outcomes over a follow-up period of up to 14 years.

**Methods:**

A nationwide, matched, longitudinal study was conducted using data from the Swedish National Diabetes Register (NDR) and the Swedish Obesity Surgery Registry (SOReg). Between 2007 and 2020, all individuals with T2DM who underwent primary surgery (RYGB = 7294 and SG = 1105) were identified through SOReg and matched by age, sex, and BMI to a control group of individuals with T2DM from NDR who had not undergone surgery (n = 8399). Data on all-cause mortality and obesity- and surgery-related outcomes after RYGB and SG were retrieved from national registers with almost complete coverage. Risks were expressed as incidence rates per 10,000 person-years and analysed using adjusted Cox regression models, which included duration of diabetes, yielding adjusted hazard ratios (HR) with 95% confidence intervals (CI).

**Findings:**

During follow-up, the percentage total weight loss and reductions in HbA1c levels were significantly greater after RYGB and SG than in unexposed individuals (%TWL: RYGB 23·2 vs. 3·6 and SG 17·1 vs. 3·1 at two years, smd > 0·1) and (mean HbA1c: RYGB 46 (SD 14) vs. 58 (SD 17) and SG 46 (SD 13) vs. 55 (SD 15) at two years, smd > 0·1). RYGB was associated with sustainable reductions in all-cause mortality (adjusted HR of 0·62 (95% CI [0·51–0·71])) and obesity-related comorbidities, with risks as much as 45% lower compared to unexposed individuals (p < 0·001). However, individuals after RYGB face as much as a twofold increased risk of malabsorption and micronutrient deficiency (adjusted HR of 2·00 (95% CI [1·76–2·28])) and alcohol use disorder (adjusted HR of 2·82 (95% CI [2·37–3·36])), p < 0·001. The risk of other psychiatric disorders, such as depression (adjusted HR of 1·28 (95% CI [1·14–1·43])), and surgical complications, such as bowel obstruction (adjusted HR of 3·96 (95% CI [3·15–4·98])), was also higher after RYGB (p < 0·001). In contrast, the SG cohort showed no significant effects on obesity-related conditions and risk of surgical complications, despite similar weight reduction in both surgery groups.

**Interpretation:**

The study highlights the advantages and limitations of RYGB and SG, providing insights to guide an individualised approach. The limited efficacy of SG in lowering obesity-related disease risks should be a key consideration when selecting individuals with T2DM for surgery.

**Funding:**

A grant from the Swedish state under the agreement with the county councils.

**Research in context**

**Evidence before this study**

We conducted a comprehensive PubMed search from the database's inception until March 31, 2025, using the following terms: “obesity OR obese” AND “diabetes mellitus OR type 2 diabetes” AND “bariatric surgery OR gastric bypass OR roux-en-Y gastric bypass OR gastric bypass OR gastrectomy OR gastric sleeve” AND “outcome OR complication.” Each search replaced “outcome OR complication” with a specific outcome assessed in this study.

Most of the studies identified were observational, with some randomised controlled trials and meta-analyses. We included studies with a follow-up of at least one year. While strong evidence supports the short-term benefits of bariatric surgery for individuals with and without diabetes, long-term data are limited. Available studies suggest that improvements in metabolic and obesity-related comorbidities persist over time. However, few studies have examined long-term safety outcomes in individuals with type 2 diabetes, particularly those extending beyond five years. This has left gaps in understanding the long-term effects of Roux-en-Y gastric bypass on individuals with type 2 diabetes, and even more so for sleeve gastrectomy. Our study aims to address these gaps by utilising nationwide registry data with extended follow-up.

**Added value of this study**

This study provides one of the most comprehensive long-term assessments of Roux-en-Y gastric bypass and sleeve gastrectomy in individuals with type 2 diabetes. Using extensive nationwide registry data with up to 14 years of follow-up, we evaluated not only obesity-related outcomes but also surgery-related complications. Our cohort includes all individuals in Sweden diagnosed with type 2 diabetes who underwent Roux-en-Y gastric bypass or sleeve gastrectomy between 2007 and 2020, compared to matched unexposed individuals with type 2 diabetes who have not undergone surgery.

Our findings confirm that RYGB leads to sustained reductions in overall mortality, cardiovascular and metabolic events, and cancer, reinforcing its effectiveness in managing obesity-related comorbidities. However, these benefits come with a higher risk of psychiatric disorders, malabsorption, micronutrient deficiency, and surgical complications requiring specialist care. In contrast, sleeve gastrectomy, despite similar effects on weight loss and improved HbA1c levels, did not significantly reduce overall mortality or the risk of cardiovascular disease, cancer, and chronic kidney disease. Although the risk of surgical-related complications was lower, sleeve gastrectomy may not be suitable in individuals with type 2 diabetes with a higher cardiovascular risk burden.

**Implications of all the available evidence**

These findings offer significant insights for clinical decision-making and future research. A holistic approach to bariatric care is essential for individuals with type 2 diabetes and obesity. Our findings support Roux-en-Y gastric bypass as the preferred option for long-term metabolic and cardiovascular benefits while emphasising the need for thorough psychiatric screening and post-surgical support, particularly regarding alcohol and substance use disorders. Furthermore, lifelong nutritional monitoring and improved strategies for better adherence to supplementation remain critical. Sleeve gastrectomy may still serve as a viable option for individuals prioritising weight loss only, but it appears less effective for those at elevated cardiovascular risk. Future research should investigate personalised treatment approaches tailored to specific risk factors and assess the role of bariatric surgery in light of newer interventions, such as incretin-based medical therapies, in the management of obesity and diabetes.

## Introduction

Bariatric surgery is a widely recognised and effective treatment option for people with obesity and type 2 diabetes (T2DM). Following bariatric surgery, individuals with T2DM experience significant long-term weight loss, and it has been reported to reduce mortality and cardiovascular risk.^1^^,^^2^ The two most common bariatric procedures, Roux-en-Y gastric bypass (RYGB) and sleeve gastrectomy (SG) have both demonstrated positive effects.^3^

Despite compelling evidence on the weight–loss benefits of RYGB and SG, both procedures are associated with risks of complications requiring hospital care.^4^ Previous studies on RYGB have shown an increased risk of malabsorption and micronutrient deficiency, deterioration of psychiatric disorders, and alcohol abuse after RYGB compared to non-surgically treated individuals.^5^, ^6^, ^7^ In a study combining data from the SLEEVEPASS and SM-BOSS trials, up to a third of participants experienced a surgical complication after undergoing RYGB or SG during the five-year follow-up.^3^ However, most previous studies of RYGB and SG included relatively few individuals with T2DM.^8^ This has left gaps in the understanding of the long-term effects of RYGB, and even more so for SG in people with T2DM.

Therefore, this observational nationwide cohort study aimed to evaluate the long–term efficacy of both RYGB and SG in T2DM, extending up to 14 years after surgery. The overall survival, obesity-related cardiovascular and metabolic comorbidities, and surgically related complications after RYGB and SG were compared to those of non-surgically treated individuals with T2DM.

## Methods

### Study design and support

This is a nationwide, registry-based, observational study of individuals with obesity and T2DM, examining the effects of RYGB and SG on long-term health compared to non-surgically treated individuals. Participants were identified by cross-matching two nationwide registries: the Swedish National Diabetes Register (NDR) and the Swedish Obesity Surgery Registry (SOReg). The present study was approved by the Ethical Review Authority in Sweden (Dn. no. 2020–05410). Individuals in NDR and SOReg have previously received information regarding data collection in these registries and its use for research purposes. Access to the datasets from registries is strictly regulated and, therefore, not publicly available.

### Data sources and study population

Since 1996, NDR has become an invaluable resource for medical information. It contains longitudinal data on approximately 90% of all individuals aged 18 years and older diagnosed with T2DM in Sweden.^9^ The data includes information on clinical characteristics, medications, risk-factor control measures such as blood pressure and biochemical values, and diabetes-related complications.^10^

In NDR, T2DM is defined using classic epidemiological criteria.^11^^,^^12^ This includes individuals treated with diet alone or in combination with oral antihyperglycemic agents. Individuals diagnosed at age 40 or older and treated with insulin, with or without oral antihyperglycemic agents, were likewise classified as having T2DM.^5^ For individuals with missing information on diabetes classification, physicians determined their diabetes status through clinical evaluation.^11^

SOReg began in 2007 and contains data on nearly all individuals undergoing bariatric surgery in Sweden.^13^ The registry provides detailed information on the type of surgery, peri- and postoperative effects, and complications up to 15 years after surgery.^13^ Individuals undergoing bariatric surgery in Sweden were selected based on clinical eligibility criteria and patient preference, in accordance with international guidelines.^14^

Individuals with T2DM who underwent RYGB or SG from January 1, 2007 to December 31, 2020, were included from SOReg. They were matched with individuals with obesity and T2DM from NDR who had not undergone surgical treatment for obesity (unexposed) at the time of matching.

All individuals were cross-matched with other national registries to obtain information on socioeconomic status, comorbidities, and pharmacotherapies. The methods section of the Supplementary Appendix provides further details on the other national registries and the cross-matching process.

### Outcomes

Outcome data were retrieved from the Swedish National Patient Registry and NDR.^15^ The following incident outcomes were analysed: all-cause mortality, cardiovascular death, ischaemic heart disease, acute coronary syndromes, cerebrovascular disease, heart failure, dysrhythmias, chronic kidney disease, diabetes-related microvascular complications, hyper– and hypoglycemia, mental health disorders, surgical–related diseases and complications such as malabsorption and micronutrient deficiency, reflux disorders and GI–ulcerations, bleeding, bowel obstruction, and hernia. Incident diagnoses were identified using coding according to the International Classification of Diseases version 10 (ICD-10), as detailed in Supplementary Table S1. Secondary outcomes, including BMI and HbA1c, were retrieved from the NDR.

### Statistical analyses

Only individuals aged 18 years or older were included in the study. Individuals who underwent surgery were matched 1:1 with unexposed individuals based on sex, date of birth, age in whole years, and BMI with one decimal, at the time of inclusion. The date of surgery served as the point of inclusion and start of follow-up for unexposed individuals. Standardised mean difference (SMD) was used to assess baseline differences, with an SMD greater than 0·1 indicating residual imbalance post-matching. The participants were followed until an outcome event, emigration, or death occurred, or else they were censored on December 31, 2021.

Incidence rates were reported as events per 10,000 person-years. Kaplan–Meier curves were used to visualise cumulative hazard estimates (1—Kaplan-Meier estimate), with SG outcomes plotted for up to 8·5 years of follow-up, as fewer than 50 individuals remained at risk beyond that point. The full number-at-risk tables are provided in the Supplementary Material (Figures S2 and S3).

Cox proportional hazards regression models were used to estimate hazard ratios (HR) with 95% confidence intervals (CI) for all outcomes. The models were adjusted for sex and baseline variables, including BMI, diabetes duration at inclusion, marital status, educational level, income quartile, smoking status, level of physical activity, and the presence of each outcome at baseline.

Competing risk analyses were deemed unnecessary due to the low mortality rate in this relatively young cohort, minimising the impact of competing risk. Therefore, standard time-to-event methods were applied, which provide valid and interpretable results in this context.

The proportional hazards assumption was evaluated using the Grambsch–Therneau test, Schoenfeld's residual plots, and smooth hazard plots. While statistical tests indicated potential violations in some models, visual inspection showed no systematic deviations, with sparse observations at the end of follow–up likely influencing the results.

Approximately 10% of unexposed individuals underwent surgery during follow–up and were included as new surgical cases, along with their matched unexposed individuals. A sensitivity analysis was conducted, excluding controls who later underwent surgery with RYGB or SG. Due to the difference in follow-up time for RYGB and SG, a sensitivity analysis was also conducted with follow-up restricted to 4 years to assess the robustness of the findings regarding all-cause mortality and major cardiovascular events.

In the SG cohort, forty individuals underwent revision to RYGB, while five individuals underwent revision to biliopancreatic diversion with duodenal switch.

Owing to the robustness of the national registries, missing data were minimal, except for income data from 2020 to 2021, which were imputed using the last observation carried forward method.

In all analyses, the surgery cohorts were compared to their matched unexposed individuals, with a two-sided significance level of 0·05. Given the risk of Type I errors, the results should be interpreted as exploratory, as no adjustment for multiple testing was made. Analyses were conducted using the R Studio statistical programming language (version 2024·12·0+467).

### Role of the funding source

The Swedish Association of Local Authorities and Regions funds NDR. The study was partially funded by grants from the Swedish state under the agreement between the Swedish government and the county councils, called the ALF agreement.

The funding sponsors were not involved in the design and conduct of the study, the analysis, writing, or the decision to submit the paper for publication.

## Results

### Study population

In total, 8399 non-randomised surgically treated individuals with T2DM and obesity, including 7294 RYGB and 1105 SG, and 8399 non–surgical treated matched unexposed individuals were included in the analyses. The flow of participants in the study is shown in Supplementary Figure S1. The clinical characteristics of RYGB and SG–treated individuals with their matched unexposed individuals are displayed in Table 1. The mean age for all four groups was 49 years (SD 10). The operated groups, RYGB and SG, had higher levels of education, income and registered civil partnerships than their unexposed individuals. The operated groups also reported lower nicotine use and engaged in less physical activity than unexposed individuals. Furthermore, the RYGB group had a lower proportion of females (58·9% vs. 63·9%) and a higher level of HbA1c (60·4 vs. 57·6 mmol/mol) compared to the unexposed individuals.

**Table 1:** Baseline characteristics for people undergoing Roux–en–Y gastric bypass or sleeve gastrectomy, and matched unexposed individuals.

	RYGB^a^ (n = 7294)	Unexposed individuals (n = 7294)	SMD^c^	SG^b^ (n = 1105)	Unexposed individuals (n = 1105)	SMD^c^
Sex = Female (%)	4298 (58·9)	4661 (63·9)	0·10	657 (59·5)	664 (60·1)	0·01
Age (mean (SD))	49·10 (9·55)	48·51 (10·20)	0·06	48·95 (9·90)	48·75 (10·42)	0·02
Body mass index (mean (SD))	41·5 (5·5)	41·0 (6·1)	0·08	40·7 (5·4)	40·6 (6·1)	0·02
Education level n (%)			**0·18**			**0·20**
Pre-secondary education ≤ 9 years	1449 (20·0)	1958 (27·4)		200 (18·2)	276 (25·6)	
Secondary education> 9–12 years	4302 (59·3)	3883 (54·3)		604 (55·0)	572 (53·0)	
Post-secondary education ≥ 12 years	1502 (20·7)	1312 (18·3)		295 (26·8)	231 (21·4)	
Civil status: married n (%)	3476 (47·7)	3147 (43·1)	**0·34**	532 (48·1)	467 (42·3)	**0·36**
Nationality = Sweden n (%)	5649 (77·4)	5353 (73·4)	0·10	776 (70·2)	755 (68·3)	0·04
Income quartiles n (%)			**0·23**			**0·28**
Quartile 1	1346 (20·4)	1841 (28·5)		162 (18·8)	232 (27·9)	
Quartile 2	1579 (23·9)	1704 (26·4)		203 (23·6)	234 (28·1)	
Quartile 3	1811 (27·4)	1496 (23·2)		249 (29·0)	184 (22·1)	
Quartile 4	1872 (28·3)	1412 (21·9)		246 (28·6)	183 (22·0)	
Smoking status n (%)	982 (13·5)	1322 (18·1)	**0·19**	150 (13·6)	195 (17·6)	**0·21**
Physical activity n (%)			**0·53**			**0·42**
1 = Rarely/never	1606 (22·0)	2209 (30·3)		268 (24·3)	335 (30·3)	
2 = 1–5 times/week	1770 (24·3)	2540 (34·8)		312 (28·2)	385 (34·8)	
3 = Daily	789 (10·8)	1143 (15·7)		151 (13·7)	205 (18·6)	
Diabetes duration (mean (SD))	6·60 (6·52)	6·19 (6·28)	0·07	5·84 (6·18)	5·75 (5·87)	0·02
Glycated haemoglobin^d^ levels (mean (SD))	60 (17)	58 (17)	**0·17**	56 (15)	56 (16)	0·05
Diastolic blood pressure (mean (SD))	80·52 (9·52)	80·57 (9·72)	0·004	81·04 (9·51)	80·69 (9·95)	0·04
Systolic blood pressure (mean (SD))	132·57 (14·19)	132·55 (15·15)	0·001	130·99 (14·09)	131·19 (14·93)	0·01
Total cholesterol (mean (SD))	4·77 (1·09)	4·83 (1·07)	0·06	1·10 (0·31)	1·12 (0·30)	0·09
High–density lipoprotein cholesterol (mean (SD))	1·10 (0·35)	1·13 (0·32)	0·09	2·84 (0·96)	2·84 (0·98)	0·001
Low–density lipoprotein cholesterol (mean (SD))	2·77 (0·95)	2·85 (0·94)	0·09	4·75 (1·11)	4·71 (1·08)	0·03
Triglycerides (mean (SD))	2·32 (1·73)	2·18 (1·48)	0·09	2·39 (1·67)	2·21 (1·74)	**0·11**
S–creatinine (mean (SD))	68·92 (34·90)	67·97 (26·80)	0·03	72·49 (59·86)	67·63 (20·09)	**0·11**
Albuminuria n (%)			**0·11**			0·10
No albuminuria	6261 (85·8)	5982 (82·0)		959 (86·8)	923 (83·5)	
Normal albuminuria	57 (0·8)	98 (1·3)		8 (0·7)	6 (0·5)	
Microalbuminuria	700 (9·6)	873 (12·0)		103 (9·3)	131 (11·9)	
Macroalbuminuria	276 (3·8)	341 (4·7)		35 (3·2)	45 (4·1)	
eGFR (mean (SD))	98·57 (25·86)	97·99 (27·28)	0·02	98·29 (27·91)	98·69 (26·92)	0·01
Cardiovascular						
Cardiovascular disease n (%)	756 (10·4)	685 (9·4)	0·03	84 (7·6)	96 (8·7)	0·04
Acute myocardial infarction n (%)	229 (3·1)	201 (2·8)	0·02	25 (2·3)	37 (3·3)	0·07
Coronary heart disease n (%)	610 (8·4)	529 (7·3)	0·04	64 (5·8)	73 (6·6)	0·03
Stroke n (%)	139 (1·9)	152 (2·1)	0·01	16 (1·4)	20 (1·8)	0·03
Atrial fibrillation/flutter n (%)	276 (3·8)	267 (3·7)	0·01	37 (3·3)	38 (3·4)	0·01
Heart failure n (%)	237 (3·2)	289 (4·0)	0·04	25 (2·3)	29 (2·6)	0·02
Hypertension n (%)	4073 (55·8)	2257 (30·9)	**0·52**	564 (51·0)	330 (29·9)	**0·44**
Venous thromboembolism n (%)	186 (2·6)	205 (2·8)	0·02	33 (3·0)	28 (2·5)	0·03
Diabetes–related disorders						
Hypoglycaemia n (%)	13 (0·2)	24 (0·3)	0·03	0 (0·0)	3 (0·3)	0·07
Hyperglycaemia n (%)	1678 (23·0)	1409 (19·3)	0·09	222 (20·1)	184 (16·7)	0·08
Diabetic microvascular complications n (%)	1544 (21·2)	1252 (17·2)	0·10	188 (17·0)	144 (13·0)	**0·11**
Psychiatric disorders						
Depression n (%)	880 (12·1)	819 (11·2)	0·03	160 (14·5)	145 (13·1)	0·04
Anxiety disorders n (%)	878 (12·0)	941 (12·9)	0·03	166 (15·0)	180 (16·3)	0·04
Alcohol use disorders n (%)	135 (1·9)	216 (3·0)	0·07	32 (2·9)	36 (3·3)	0·02
Other drug use disorders n (%)	126 (1·7)	160 (2·2)	0·03	29 (2·6)	21 (1·9)	0·05
Suicide attempt	0	0	n. a	0	0	n. a
Other diseases						
Chronic kidney disease n (%)	150 (2·1)	166 (2·3)	0·02	51 (4·6)	25 (2·3)	0·13
Malabsorption and micronutrient deficiency n (%)	243 (3·3)	209 (2·9)	0·03	42 (3·8)	35 (3·2)	0·04
Cancer n (%)	339 (4·6)	420 (5·8)	0·05	71 (6·4)	71 (6·4)	<0·001
Pulmonary disease n (%)	818 (11·2)	660 (9·0)	0·07	126 (11·4)	91 (8·2)	0·11
Gastrointestinal disorders						
Hernia n (%)	603 (8·3)	444 (6·1)	0·09	109 (9·9)	82 (7·4)	0·09
Gastrointestinal reflux and ulcer n (%)	455 (6·2)	317 (4·3)	0·09	71 (6·4)	44 (4·0)	**0·11**
Bowel obstruction n (%)	24 (0·3)	32 (0·4)	0·02	10 (0·9)	6 (0·5)	0·04
Abdominal pain n (%)	1466 (20·1)	1413 (19·4)	0·02	315 (28·5)	250 (22·6)	**0·14**
Gastrointestinal leakage n (%)	16 (0·2)	21 (0·3)	0·01	4 (0·4)	5 (0·5)	**0·01**
Liver disease n (%)	100 (1·4)	87 (1·2)	0·02	29 (2·6)	23 (2·1)	0·04
Gallbladder and pancreatic diseases n (%)	537 (7·4)	493 (6·8)	0·02	93 (8·4)	71 (6·4)	0·08
Prescription drugs						
Antihyperglycemic n (%)	6767 (94·0)	6251 (89·0)	0	1019 (94·0)	987 (92·0)	0·10
Anticoagulant agents n (%)	3052 (42·4)	1775 (25·3)	0	388 (36·6)	180 (16·7)	**0·33**
Calcium and/or vitamin D supplements n (%)	5832 (80·0)	627 (8·6)	**2·07**	847 (76·7)	75 (6·8)	**2·01**
Anaemia medication	1993 (27·7)	1082 (15·4)	0	283 (26·0)	183 (17·0)	**0·19**
Gastrointestinal agents n (%)	4335 (59·4)	2323 (31·8)	**0·58**	962 (87·1)	342 (31·0)	**1·40**
Weight–loss agents (%)	76 (1·0)	223 (3·1)	**0·14**	6 (0·5)	21 (1·9)	**0·12**
Antihypertensive agents n (%)	4742 (65·0)	4426 (60·7)	0·09	681 (61·6)	630 (57·0)	0·09
Hypolipidemic agents n (%)	2761 (37·9)	3713 (50·9)	**0·27**	432 (39·1)	577 (52·2)	**0·27**
Antidepressant agents n (%)	1845 (25·3)	1664 (22·8)	0·06	296 (26·8)	266 (24·1)	0·06
Sedatives and hypnotics n (%)	1662 (22·8)	1497 (20·5)	0·06	276 (25·0)	246 (22·3)	0·06

Unexposed individuals are individuals matched for sex, age, and body mass index who were selected from the National Diabetes Registry.

Bold values indicate statistical significance according to SMD (>0·10).

a RYGB = Roux–en–Y Gastric bypass.

b SG = Sleeve gastrectomy.

c SMD = Standardised mean difference, SMD > 0·1 is considered statistically significant.

d Concentrations of glycated haemoglobin (HbA1c) are based on values from the International Federation of Clinical Chemistry, unit mmol/mol.

At baseline, hypertension was the only comorbidity more prevalent in the RYGB and SG groups (Table 1); however, antihypertensive prescription did not differ from that of unexposed individuals. All surgical cases receive calcium and vitamin D supplementation postoperatively, and many of these prescriptions are prepared preoperatively, explaining the higher portion of baseline supplementation compared to unexposed individuals (Table 1). Additional diagnoses and prescribed drugs are included in Supplementary Table S3.

The median follow-up time was 9 years for the RYGB cohort and 4 years for the SG cohort, including their respective control groups.

### Incidence rates, all-cause mortality, and risk of obesity- and surgical-related disease after Roux-en-Y gastric bypass

**Fig. 1:**
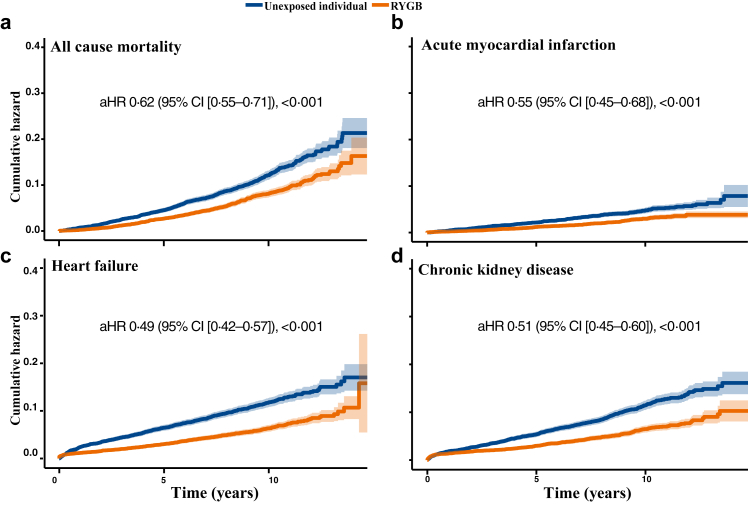
**Accumulated risk of obesity-related diseases in individuals with type 2 diabetes and obesity following Roux-en-Y gastric bypass compared to matched unexposed individuals.** A Cox model was constructed for each outcome to estimate the cumulative hazard associated with RYGB compared to matched unexposed individuals (Panels a–d). The lines represent the adjusted hazard function (aHR) and the shaded area 95% confidence intervals (CI).

**Fig. 2:**
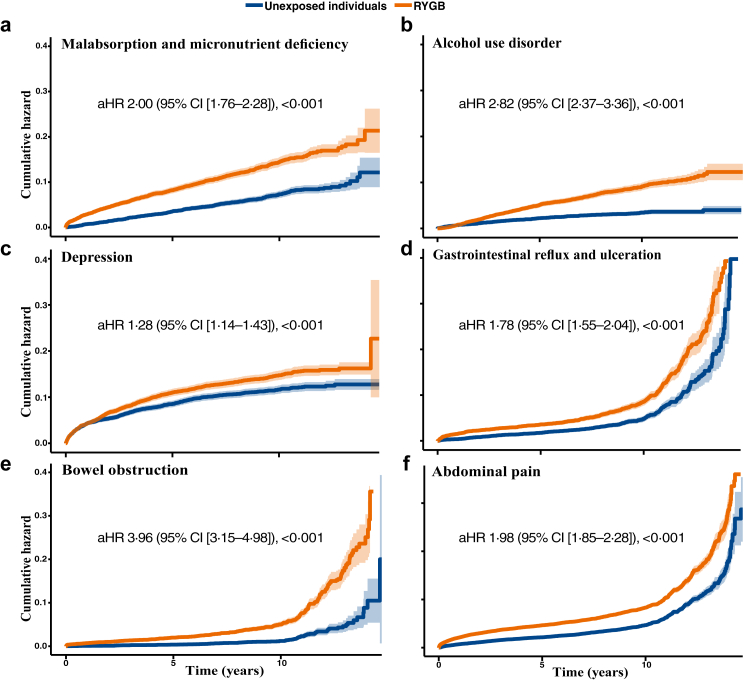
**Accumulated risk of obesity-related diseases in individuals with type 2 diabetes and obesity following Roux-en-Y (RYGB) gastric bypass compared to matched unexposed individuals.** A Cox model was constructed for each outcome to estimate the cumulative hazard associated with RYGB compared to matched unexposed individuals (Panels a–f). The lines represent the adjusted hazard (aHR) function and the shaded area 95% confidence intervals (CI).

Results for incidence rates per 10,000 person-years and the adjusted hazard ratios are presented in Table 2, with cumulative hazard curves for selected outcomes in Fig. 1 Panel a–d and Fig. 2 Panel a–f. To avoid visual overcrowding in Fig. 1 Panel a–d and Fig. 2 Panel a–f, the corresponding number-at-risk tables are presented in the Supplementary Material (Figure S2). The incidence of all-cause mortality was 78·8 per 10,000 person-years (95% CI [71·9–86·2]) in the RYGB cohort and 119·1 per 10,000 person-years (95% CI [110·5–128·2]) in the unexposed control group, with an adjusted HR of 0·62 (95% CI [0·51–0·71]) (Fig. 1 Panel a and Table 2). Similar results were found for acute myocardial infarction (Fig. 1 Panel b), heart failure (Fig. 1 Panel c), stroke, chronic kidney disease (Fig. 1 Panel d), and microvascular diabetic complications with lower rates after RYGB compared to unexposed individuals (Table 2).

**Table 2:** Incidence rates per 10,000 person-years and adjusted hazard ratios for obesity- and surgical-related outcomes after Roux-en-Y gastric bypass compared with unexposed individuals.

	RYGB^a^ (n = 7294)	Unexposed individuals (n = 7294)	aHR [95% CI]^b^	p-value
All-cause mortality	78·8 [71·9–86·2]^c^	119·1 [110·5–128·2]	0·62 [0·55–0·71]	<0·001
Cardiovascular diseases				
Fatal cardiovascular disease	22·7 [19·1–26·9]	42·5 [37·4–48·1]	0·50 [0·40–0·63]	<0·001
Cardiovascular disease	171·6 [160·8–182·8]	194·4 [182·8–206·5]	0·88 [0·79–0·97]	0·008
Acute myocardial infarction	27·9 [23·8–32·4]	47·1 [41·7–53·0]	0·55 [0·45–0·68]	<0·001
Coronary heart disease	126·6 [117·4–136·2]	138·8 [129·1–149·0]	0·91 [0·81–1·01]	0·08
Heart failure	65·4 [59·0–72·3]	121·4 [112·4–130·8]	0·49 [0·42–0·57]	<0·001
Stroke	39·8 [34·9–45·2]	48·9 [43·3–54·9]	0·78 [0·64–0·94]	0·008
Atrial fibrillation/flutter	95·3 [87·5–103·6]	109·6 [101·2–118·6]	0·78 [0·69–0·89]	<0·001
Other dysrhythmias	123·3 [114·3–132·7]	141·9 [132·1–152·1]	0·74 [0·66–0·83]	<0·001
Valvular heart disease	23·1 [19·4–27·3]	25·7 [21·8–30·2]	0·79 [0·61–1·02]	0·067
Hypertension	642·7 [619·2–666·9]	816·1 [788·5–844·5]	0·58 [0·55–0·62]	0·001
Venous thromboembolism	38·4 [33·6–43·8]	52·6 [46·9–58·9]	0·72 [0·60–0·88]	<0·001
Diabetes-related disorders				
Hypoglycaemia	8·6 [6·5–11·3]	8·8 [6·6–11·6]	1·14 [0·74–1·76]	0·547
Hyperglycaemia	294·3 [280·0–309·1]	443·6 [425·5–462·3]	0·59 [0·55–0·63]	<0·001
Diabetic microvascular complications	282·1 [268·1–296·6]	410·8 [393·4–428·7]	0·60 [0·56–0·65]	<0·001
Psychiatric disorders				
Depression	163·5 [153·0–174·6]	130·5 [121·1–140·5]	1·28 [1·14–1·43]	<0·001
Anxiety disorders	184·6 [173·4–196·4]	156·2 [145·9–167·1]	1·38 [1·24–1·53]	<0·001
Alcohol use disorders	92·8 [85·1–101·0]	35·7 [31·0–40·9]	2·82 [2·37–3·36]	<0·001
Other drug use disorders	42·0 [37·0–47·6]	25·6 [21·6–30·0]	2·16 [1·72–2·72]	<0·001
Other diseases				
Chronic kidney disease	64·1 [57·8–70·9]	113·5 [104·9–122·6]	0·51 [0·45–0·60]	<0·001
Malabsorption and micronutrient deficiency	149·8 [139·8–160·2]	72·3 [65·5–79·6]	2·00 [1·76–2·28]	<0·001
Anaemia	188·9 [177·7–200·7]	124·8 [115·7–134·4]	1·50 [1·35–1·67]	<0·001
Cancer	99·3 [91·4–107·8]	123·7 [114·7–133·3]	0·79 [0·70–0·90]	<0·001
Pulmonary disease	94·3 [86·5–102·5]	154·7 [144·4–165·5]	0·51 [0·45–0·58]	<0·001
Osteoporosis	17·0 [13·9–20·7]	11·4 [8·8–14·5]	1·35 [0·95–1·90]	0·093
Fractures	175·3 [164·5–186·6]	96·9 [88·9–105·3]	1·82 [1·62–2·04]	<0·001
Gastrointestinal diseases				
Hernia	145·9 [136·0–156·3]	93·2 [85·4–101·6]	1·42 [1·26–1·61]	<0·001
Gastrointestinal reflux and ulcer	107·8 [99·5–116·5]	62·4 [56·2–69·2]	1·78 [1·55–2·04]	<0·001
Abdominal pain	507·7 [488·1–527·8]	280·8 [266·9–295·3]	1·98 [1·85–2·13]	<0·001
Non–alcoholic fatty liver disease	9·5 [7·2–12·3]	16·2 [13·1–19·8]	0·57 [0·40–0·83]	0·002
Gallbladder and pancreatic diseases	146·4 [136·5–156·7]	80·4 [73·2–88·1]	1·85 [1·63–2·11]	<0·001
Surgical complications				
Bowel obstruction	68·2 [61·7–75·1]	17·8 [14·6–21·6]	3·96 [3·15–4·98]	<0·001
Gastrointestinal leakage	20·8 [17·3–24·8]	9·7 [7·3–12·5]	2·27 [1·61–3·20]	<0·001
Surgical wound complications	53·7 [47·9–59·9]	30·2 [25·9–35·0]	1·97 [1·61–2·40]	<0·001

a RYGB = Roux-en-Y Gastric Bypass.

b aHR = adjusted Hazard Ratio, CI = Confidence interval, between RYGB and unexposed individuals.

c Incidence rates per 10,000 person-years with confidence intervals.

The incidence of malabsorption and micronutrient deficiency was higher after RYGB (149·8, 95% CI [139·8–160·2]) compared to the unexposed individuals (72·3, 95% CI [65·5–79·6]), with an adjusted HR of 2·00 (95% CI [1·76–2·28]) (Fig. 2 Panel a and Table 2). Additionally, RYGB was associated with a significantly higher incidence of fractures (adjusted HR 1·82, 95% CI [1·62–2·04]) and anaemia (adjusted HR 1·50, 95% CI [1·35–1·67]) during the follow–up. The incidence of alcohol use disorder was higher after RYGB with an adjusted HR of 2·82 (95% CI [2·37–3·36]) (Fig. 2 Panel b and Table 2). The rates of depressive (Fig. 2 Panel c) and anxiety disorders, as well as drug addiction, were significantly higher after RYGB. The incidence rates and adjusted HRs for these outcomes are reported in Table 2.

RYGB was associated with a significantly higher incidence of abdominal pain (adjusted HR 1·98, [1·85–2·13]) (Fig. 2 Panel f), gallstone and pancreatic disease (adjusted HR 1·85, 95% CI [1·63–2·11]) and gastrointestinal reflux and ulceration (62·4 vs. 107·8 per 10,000 person-years, adjusted HR 1·78 (95% CI [1·55–2·04])) (Fig. 2 Panel d). Surgical complications were more common after RYGB, with bowel obstruction being four times more likely compared to unexposed individuals (adjusted HR 3·96, 95% CI [3·15–4·98]) (Fig. 2 Panel e). The incidence of GI leakage was also higher following RYGB (adjusted HR 2·27, 95% CI [1·61–3·20]).

A sensitivity analysis excluding unexposed individuals who later underwent RYGB, along with their matched surgical cases, yielded results consistent with the main findings (Supplementary Table S8).

### Incidence rates, all-cause mortality, and risk of obesity- and surgical-related disease after sleeve gastrectomy

**Fig. 3:**
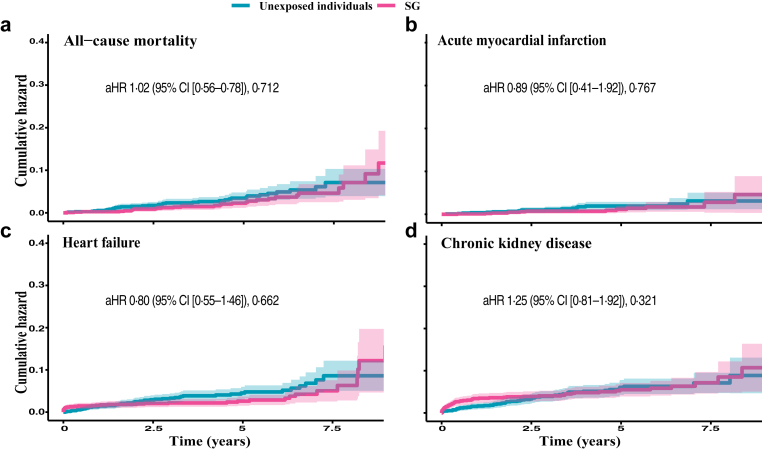
**Accumulated risk of obesity-related diseases in individuals with type 2 diabetes and obesity following sleeve gastrectomy (SG) compared to matched unexposed individuals.** A Cox model was constructed for each outcome to estimate the cumulative hazard associated with SG compared to matched unexposed individuals (Panels a–d). The lines represent the adjusted hazard (aHR) function and the shaded area 95% confidence intervals (CI).

**Fig. 4:**
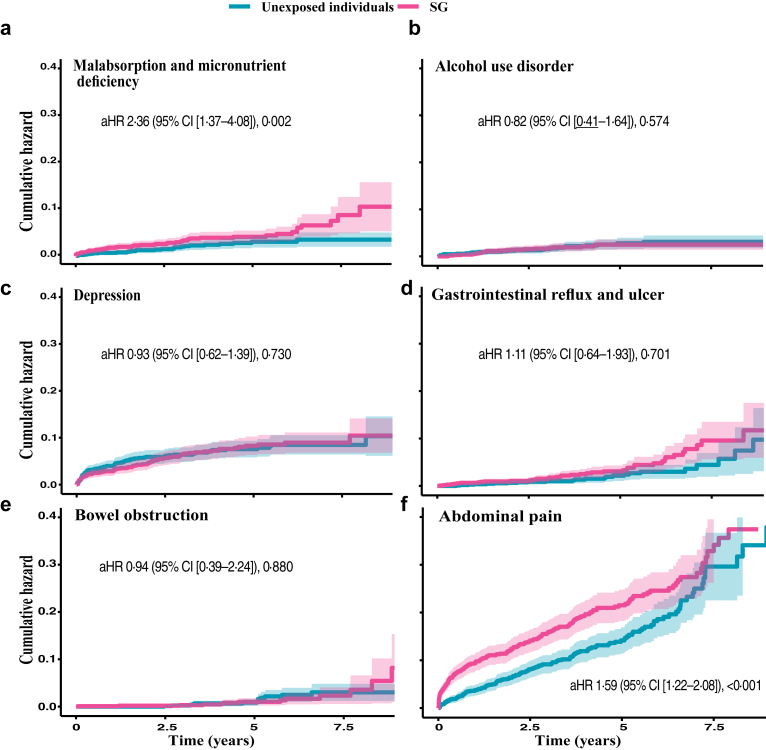
**Accumulated risk of obesity-related diseases in individuals with type 2 diabetes and obesity following sleeve gastrectomy (SG) compared to matched unexposed individuals.** A Cox model was constructed for each outcome to estimate the cumulative hazard associated with SG compared to matched unexposed individuals (Panels a–f). The lines represent the adjusted hazard (aHR) function and the shaded area 95% confidence intervals (CI).

Long-term incidence rates per 10,000 person-years and adjusted hazard ratios are presented in Table 3, with cumulative hazard curves for selected outcomes in Fig. 3 Panel a–d and Fig. 4 Panel a–f. To avoid visual overcrowding in Fig. 3 Panel a–d and Fig. 4 Panel a–f, the corresponding number-at-risk tables are presented in the Supplementary Material (Figure S3). The overall all-cause mortality rate was 63·0 per 10,000 person-years (95% CI [42·8–89·4]) for the SG cohort and 77·9 per 10,000 person-years (95% CI [55·1–106·9]) for the control group, with an adjusted HR of 1·02 (95% CI [0·58–1·78]) (Fig. 3 Panel a and Table 3). The incidence rates of coronary heart disease, hypertension, and microvascular diabetic complications were significantly lower following SG compared to the control group (Table 3).

**Table 3:** Incidence rates and adjusted hazard ratios for obesity– and surgical–related outcomes after sleeve gastrectomy compared with matched unexposed individuals.

	SG^a^ (n = 1105)	Unexposed individuals (n = 1105)	aHR [95% CI]^b^	p-value
All-cause mortality	63·0 [42·8–89·4]	77·9 [55·1–106·9]^c^	1·02 [0·58–1·78]	0·712
Cardiovascular disease				
Cardiovascular disease	191·2 [153·5–235·3]	185·9 [148·7–229·6]	1·42 [1·01–1·90]	0·046
Fatal cardiovascular disease	12·2 [4·5–26·5]	26·7 [14·2–45·6]	0·52 [0·16–1·69]	0·275
Acute myocardial infarction	28·6 [15·6–48·0]	37·2 [22·1–58·8]	0·89 [0·41–1·92]	0·767
Coronary heart disease	148·6 [115·8–187·7]	136·7 [105·3–174·6]	1·60 [1·08–2·37]	0·018
Heart failure	74·6 [52·2–103·3]	98·4 [72·3–130·8]	0·80 [0·55–1·46]	0·662
Stroke	32·7 [18·7–53·2]	47·7 [30·2–71·5]	0·75 [0·36–1·59]	0·458
Atrial fibrillation/flutter	100·4 [74·0–133·1]	133·9 [102·9–171·4]	1·01 [0·66–1·54]	0·979
Other dysrhythmias	113·2 [85·0–147·7]	155·7 [122·1–195·8]	0·78 [0·53–1·15]	0·211
Valvular heart disease	20·5 [9·8–37·7]	20·6 [9·9–37·9]	1·27 [0·40–3·25]	0·61
Hypertension	670·9 [593·6–755·6]	805·1 [718·5–899·3]	0·66 [0·54–0·79]	<0·001
Venous thromboembolism	49·6 [31·8–73·8]	58·2 [38·7–84·1]	1·04 [0·57–1·89]	0·91
Diabetes-related disorders				
Hypoglycaemia	6·1 [1·3–17·8]	8·2 [2·2–21·0]	0·54 [0·11–2·68]	0·448
Hyperglycaemia	327·2 [276·6–384·4]	501·3 [437·2–572·1]	0·62 [0·49–0·78]	<0·001
Diabetic microvascular complications	315·3 [265·7–371·4]	459·5 [398·4–527·2]	0·66 [0·51–0·84]	<0·001
Psychiatric disorders				
Depression	166·3 [131·2–207·8]	166·9 [131·7–208·6]	0·93 [0·62–1·39]	0·730
Anxiety disorders	229·3 [187·6–277·6]	224·5 [183·0–272·5]	1·28 [0·92–1·79]	0·143
Alcohol use disorders	45·4 [28·5–68·7]	49·8 [31·9–74·1]	0·82 [0·41–1·64]	0·574
Other drug use disorders	43·2 [26·7–66·0]	20·6 [9·9–37·9]	2·72 [1·10–6·74]	0·031
Other diseases				
Chronic kidney disease	120·2 [91·1–155·8]	113·8 [85·5–148·5]	1·25 [0·81–1·92]	0·321
Malabsorption and micronutrient deficiency	98·8 [72·5–131·2]	54·1 [35·4–79·3]	2·36 [1·37–4·08]	0·002
Anaemia	95·6 [70·0–127·6]	90·6 [65·6–122·1]	1·35 [0·84–2·16]	0·219
Cancer	123·0 [93·7–158·7]	138·2 [106·7–176·1]	0·94 [0·63–1·39]	0·75
Pulmonary disease	85·6 [61·4–116·1]	163·0 [128·4–204·0]	0·37 [0·24–0·58]	<0·001
Osteoporosis	8·1 [2·2–20·9]	10·3 [3·3–24·0]	0·68 [0·14–3·36]	0·636
Fractures	121·9 [92·6–157·6]	81·9 [58·3–112·0]	1·64 [1·03–2·59]	0·035
Gastrointestinal diseases				
Hernia	158·4 [124·4–198·9]	102·8 [76·1–136·0]	1·52 [0·90–2·30]	0·051
Gastrointestinal reflux and ulcer	92·4 [67·4–123·7]	51·5 [33·4–76·1]	1·11 [0·64–1·93]	0·701
Abdominal pain	488·3 [424·6–558·8]	325·8 [275·3–383·0]	1·59 [1·22–2·08]	0·001
Non–alcoholic fatty liver disease	20·4 [9·8–37·6]	31·0 [17·3–51·1]	0·44 [0·17–1·10]	0·079
Gallbladder and pancreatic diseases	136·8 [105·6–174·4]	88·6 [63·8–119·7]	1·33 [0·85–2·10]	0·218
Surgical complications				
Bowel obstruction	34·6 [20·1–55·4]	26·7 [14·2–45·6]	0·94 [0·39–2·24]	0·880
Gastrointestinal leakage	18·3 [8·4–34·8]	10·3 [3·3–23·9]	1·28 [0·36–4·62]	0·703
Surgical wound complications	53·6 [35·0–78·5]	28·8 [15·7–48·3]	1·24 [0·61–2·52]	0·557

a SG = Sleeve gastrectomy.

b aHR = adjusted Hazard Ratio, CI = Confidence interval.

c Incidence rates per 10,000 person-years with confidence intervals.

Malabsorption and micronutrient deficiencies were higher after SG (98·8, 95% CI [72·5–131·2]) compared to the unexposed individuals (54·1, 95% CI [35·4–79·3]), with an adjusted HR of 2·36 (95% CI [1·37–4·08]) (Fig. 4 Panel a and Table 3). After SG, a higher incidence of fractures (adjusted HR 1·64, [1·03–2·59]) was observed during follow–up. There was a statistically higher incidence of drug use after SG (adjusted HR 2·72, 95% CI [1·10–6·74]). The adjusted HRs for alcohol use disorder (Fig. 4 Panel b), depression (Fig. 4 Panel c), or anxiety disorders were not statistically significant (Table 3).

A higher incidence of gastrointestinal reflux and ulcerations was observed compared to the control group (92·4 vs. 51·5 per 10,000 person-years). Still, the hazard ratio did not reach statistical significance (adjusted HR 1·11, 95% CI [0·64–1·93]) (Fig. 4 Panel d and Table 3). Inpatient and specialist outpatient care for abdominal pain was significantly higher after SG compared to the unexposed individuals (Fig. 4 Panel f).

The incidence rates of surgical-related complications were higher after SG, but none of the HRs reached statistical significance (Fig. 4 Panel e and Table 3).

A sensitivity analysis excluding unexposed individuals who later underwent SG, along with their matched surgical cases, yielded results consistent with the main findings (Supplementary Table S9).

There was no significant change in the adjusted hazard ratios for RYGB or SG when the follow-up time was restricted to 4 years for all-cause mortality and major cardiovascular events (not shown).

Supplementary Tables S4 and S5 present incidence rates for the additional diagnoses included in this study. Supplementary Tables S6 and S7 present crude incidence rates for all outcomes for both study groups.

### Weight loss and HbA1c at the two-year follow-up

At two years, the percentage total weight loss (%TWL) and reduction in HbA1c levels were significantly greater after RYGB and SG than in unexposed individuals (SMD > 0·1). The RYGB group had a %TWL of 23·2 and a mean HbA1c of 46 (SD 14) mmol/mol, while unexposed individuals had a %TWL of 3·6 and HbA1c of 58 (SD 17). The SG group demonstrated a %TWL of 17·1 and mean HbA1c of 46 (SD 13), compared to unexposed individuals with a %TWL of 3·1 and HbA1c of 55 (SD 15) mmol/mol. At three and seven years, both weight-loss and HbA1c showed a modest decline, with slight weight regain and rising HbA1c observed in both the RYGB and SG cohorts (Supplementary Table S11).

## Discussion

In this nationwide, matched, registry-based cohort study, the long–term effects and safety of RYGB and SG in individuals with T2DM were compared to non–surgical unexposed individuals with obesity and T2DM. To our knowledge, this is the first large-scale study examining the positive and negative effects of RYGB and SG in people with T2DM over a follow-up period of up to 14 years. RYGB was associated with sustained reductions in overall mortality, cardiovascular and metabolic events, and cancer. However, these benefits came with an increased risk of psychiatric disorders, malabsorption and micronutrient deficiency, and surgical complications. In contrast, while SG was associated with weight loss and improved HbA1c levels, no significant long–term reductions in overall mortality and cardiovascular disease were observed.

An important observation in this study was the higher incidence of malabsorption and micronutrient deficiency following RYGB and SG compared to the unexposed individuals.^5^^,^^16^ Individuals with obesity and T2DM are already at increased risk of micronutrient deficiencies, such as vitamin D and thiamine, and this can worsen after bariatric surgery.^17^, ^18^, ^19^ A 2019 study by Liakopoulos et al. reported a twofold increased risk of malabsorption and anaemia up to 9 years after RYGB in people with T2DM, based on data from the SOReg registry.^5^ Consistent with these findings, the present study observed similar elevated risk of micronutrient deficiency up to 14 years after RYGB, along with a more than 50% increased risk of anaemia, osteoporosis, and fractures. While the risk of micronutrient deficiencies has previously been partly attributed to altered absorption mechanisms, particularly after RYGB,^20^ more recent studies suggest that factors such as preexisting nutritional deficiencies, reduced energy and nutrient intake,^21^ and inconsistent supplementary use may play a greater role.^19^^,^^22^^,^^23^ Furthermore, insufficient levels of micronutrients, such as vitamin D and magnesium, could potentially influence both diabetes remission and relapse in post-bariatric individuals.^17^ Ensuring optimised nutritional status before and after bariatric surgery, through adequate supplementation and improving adherence strategies, is essential in this group.

Another important observation of this study was the long–term burden of psychiatric disorders in this study, with higher incidences of depression, anxiety and, particularly, alcohol use disorders (AUD) persisting over time. Similar to the current study, Liakopoulos et al. also reported a threefold increased risk of AUD following RYGB in the 2019 study. These results are consistent with another Swedish cohort study, but in individuals without diabetes.^7^ Furthermore, two observational studies from the United States (US) found a twofold higher risk of AUD after RYGB compared to other bariatric procedures, including SG.^24^^,^^25^ In one of these studies, Mahmud et al. also reported a diminishing mortality benefit associated with RYGB among individuals with increased alcohol use.^25^ The elevated risk of AUD is likely multifactorial, with physiological and metabolic alterations post-RYGB affecting alcohol sensitivity post-surgery as a primary contributor.^7^^,^^24^ The current study found no increased risk for AUD following SG.^25^ However, the higher incidence of other substance use disorders suggests that long-term follow-up should include psychological monitoring regardless of the surgical procedure. The present results on depression align with previous research in individuals without diabetes.^7^^,^^26^ Unlike AUD, depression and anxiety have been associated with their pre-surgical prevalence.^26^^,^^27^ While baseline rates remained unchanged post-RYGB in the current study, the risk of specialist care for these conditions was 30–40% higher than in the control group, suggesting a potential deterioration over time, as reported in other studies.^27^, ^28^, ^29^ Additionally, 31 suicide attempts were recorded after RYGB, compared to none in the control group and one after SG. Although not statistically significant, this highlights the potential for serious psychiatric consequences following RYGB. These findings emphasise the importance of a more individualised approach to bariatric care, integrating robust mental health support into long-term follow-up for those at risk.

Specialist care for gastrointestinal disorders was common during follow-up, particularly after RYGB. The higher incidence of GI reflux and ulcers likely reflects the risk of ulcer formation associated with RYGB, which may also contribute to the increased risk of gastrointestinal leakage. The composite endpoint could explain the lack of difference in reflux and ulcer outcomes after SG. Additionally, gallstone disease was more frequent, likely due to rapid weight loss altering cholesterol and bile salt metabolism, thereby promoting gallstone formation.^8^

While RYGB has long been regarded as one of the standard bariatric procedures, SG has become increasingly common over the past decade.^30^^,^^31^ SG gained popularity worldwide due to promising long–term weight loss, technical simplicity, and notable improvements in glycaemic control.^3^^,^^31^^,^^32^ However, the present findings indicate that SG does not yield comparable benefits. Although there were lower incidences of diabetes-related microvascular and hyperglycaemic complications, no significant effect on overall mortality, cardiovascular outcomes, cancer, and chronic kidney failure was observed compared to the unexposed cohort. Previous studies with follow–up periods of up to five years have indicated that while SG leads to substantial weight loss, the overall benefits of important long-term outcomes remain inferior to those of RYGB.^33^^,^^34^ In contrast to the present study, Aminian et al. found that, although SG was inferior to RYGB, it significantly reduced major cardiovascular events compared to a non-surgical cohort with T2DM.^34^ The smaller sample size, higher baseline rates of comorbidity, and shorter follow–up in that study may explain these differences from our study.^34^

Our results suggest that RYGB is associated with more favourable outcomes than SG in terms of mortality and cardiometabolic disease, despite similar weight loss. This aligns with recent randomised controlled trials (RCTs), such as the Oseberg and SleeveBypass 5-year trials, which found greater metabolic risk reduction with RYGB, and the SM-BOSS 10-year trial, which demonstrated RYGB's superiority over SG in terms of weight loss durability.^35^, ^36^, ^37^ These RCTs reinforce real-world patterns seen in this national cohort and highlight the differing long-term profiles of the two procedures.

Several limitations must be acknowledged. First, as with all observational studies, residual confounders may linger despite high–quality registries and adjustments for imbalances that remain after matching. Although BMI and several other variables were available longitudinally, we relied on baseline values to maintain analytical consistency and reduce model complexity. This may introduce residual confounding, particularly concerning BMI, which can influence long-term outcomes. Another limitation is the incomplete follow-up data for weight and HbA1c beyond 2 years, especially in the SG cohort. This affects the ability to fully evaluate long-term metabolic durability. Furthermore, detailed follow–up data on lifestyle factors, including dietary habits and adherence to postoperative care and supplementation, were lacking, making it difficult to account for their influence. The introduction of the 2015 Scandinavian nutritional supplementation guidelines may also affect the results, as those treated after that time are more likely to have received higher, and more standardised doses of micronutrients. Second, variability in SG surgical techniques across centres may influence the findings, particularly regarding surgical complications, but should not affect the overall results. Changes in technique over time for both RYGB and SG may also influence the results. Additionally, SG becoming more common in later years may limit the generalisability of the finding to current practice. Third, approximately 10% of the unexposed cohort later underwent bariatric surgery; however, a sensitivity analysis excluding controls who later underwent bariatric surgery, along with their matched surgical cases, yielded results consistent with the main findings, suggesting that crossover had minimal impact on the overall conclusions (Supplementary Tables S8 and S9). Fourth, violations of the proportionality hazards assumption in some SG and RYGB models may have occurred due to the limited number of observations, particularly at the end of the follow-up period. Supplementary Figures S2 and S3 include numbers at risk for all-cause mortality to aid in assessing the robustness of the results. Fifth, reliance on ICD codes to assess morbidities at baseline and hard-endpoint outcomes may introduce misclassification or underreporting of diagnoses. However, this is likely minimal, as Swedish national health registries have demonstrated high validity,^15^ and diagnostic errors are unlikely to differ between the study groups. Moreover, only events requiring specialist care were included, which enhances validity but may result in underreporting due to the lack of nationwide primary care data. Sixth, baseline differences between the surgery groups and their unexposed counterparts indicate a possible selection bias in treatment allocation. Additionally, the relatively small SG group and possible differences in surgical candidacy criteria may reflect unmeasured clinical judgement or variation in disease severity, which could influence comparative outcomes. We attempted to address these biases through adjusted analyses. Finally, while the study design enhances statistical power by using large control groups, it does not allow for direct comparison between surgical methods. Although the study groups were sufficiently large to detect differences, the shorter follow-up time in the SG cohort, combined with fewer events, warrants cautious interpretation when comparing findings across the two procedures. However, a sensitivity analysis restricted to 4 years of follow-up revealed no significant change in the adjusted hazard ratios for all-cause mortality and major cardiovascular events.

The study highlights the advantages and limitations of RYGB and SG, offering insights to guide an individualised approach to bariatric treatment. RYGB continues to influence cardiovascular and metabolic health positively. However, its benefits must be weighed against notable adverse effects on GI health, nutritional status, and psychiatric well–being. The limited efficacy of SG on mortality and obesity-related outcomes is a significant finding that must be considered when selecting individuals with obesity and type 2 diabetes for surgery. SG may be appropriate for individuals with obesity alone, prioritising weight loss. Future studies with detailed analyses of different subgroups and specific diagnoses are currently being planned.

## Contributors

The study was conceptualised by the last author, BE. BE and HC had full access to the data and have verified it. BE, AM, HC, and GH designed the study. AM and HC conducted the analyses. AM wrote the first draft of the manuscript and prepared the figures and tables. All authors supported the interpretation of the findings and significantly contributed to editing the manuscript. All authors vouch for the accuracy and completeness of the data and analyses and made the decision to submit the manuscript for publication. All named authors take responsibility for the integrity of the work as a whole and have given their approval for this version to be published.

## Data sharing statement

The data used in this study are from the national registries stated in this paper. Access to the datasets used in this study is restricted due to the sensitive nature of the data. Requests for access can be made to the data providers listed in the paper in accordance with the legal and regulatory requirements in Sweden. Furthermore, access to individual-level health registry data, as used in this study, can only be approved by the Swedish Ethical Review Authority.

## Declaration of interests

AM has nothing to disclose. HC has nothing to disclose. KEO reports personal lecture fees and/or honoraria for consulting (payment to institution) from Sanofi, Eli Lilly, Novo Nordisk, AstraZeneca, and Abbott Diabetes Care. ML reports personal lecture fees and/or honoraria for consulting from Eli Lilly and Novo Nordisk. IL has nothing to disclose. JO is the director of the Scandinavian Obesity Surgery Registry (SOReg – payment for this work is made to the institution). ES reports receiving reimbursement for lectures from MSD and consultant fees from Johnson & Johnson Medical (paid to the institution). ES also reports receiving grants from Region Örebro County, Åke Wiberg Foundation, and Bengt Ihre Foundations (all paid to the institution). GH reports receiving reimbursements for lectures from Novo Nordisk, AstraZeneca, and Boehringer Ingelheim. BE reports consulting fees or payment or honoraria for lectures, presentations, speaker bureaus, manuscript writing or educational events from AMGEN, Novo Nordisk, Sanofi, Eli Lilly, Boehringer Ingelheim, AstraZeneca, and MSD. All reports of reimbursements were for work unrelated to the current study's content. The authors declare that the current research was conducted without any commercial or financial relationships that could be interpreted as potential conflicts of interest.
