# Supplementary material for: Long–term effects of gastric bypass and sleeve gastrectomy in type 2 diabetes: a matched retrospective cohort study from Sweden
Source: Lancet Reg Health Eur. 2025 Aug 30;58:101430. doi: 10.1016/j.lanepe.2025.101430 (PMC12418880; doi:10.1016/j.lanepe.2025.101430)
Supplement: Supplementary Materials [file mmc1.docx]

**SUPPLEMENTAL APPENDIX**

Table of Contents

[Methods 2](#_Toc199544128)

[Data sources 2](#_Toc199544129)

[Supplementary tables 3](#_Toc199544130)

[Table S1. ICD–codes used to define baseline comorbidities up to 10 years before the index date and outcomes during follow–up 2007–2021. 3](#_Toc199544131)

[Table S2. ATC–codes used to define baseline prescription therapies. 5](#_Toc199544132)

[Table S3. Continuation of Table 1: baseline comorbidities and prescription drugs. 6](#_Toc199544133)

[Table S4. Continuation of Table 2: Incidence rates and adjusted Hazard ratios for each outcome after Roux–en–Y Gastric Bypass compared with matched unexposed individuals. 8](#_Toc199544134)

[Table S5. Continuation of Table 3: Incidence rates and adjusted Hazard ratios for each outcome after Sleeve Gastrectomy compared with matched unexposed individuals. 9](#_Toc199544135)

[Table S6. Number of events and person–years for each outcome after Roux–en–Y Gastric Bypass compared with matched unexposed individuals. 10](#_Toc199544136)

[Table S7. Number of events and person–years for each outcome after Sleeve Gastrectomy compared with matched unexposed individuals. 12](#_Toc199544137)

[Table S8. Outcomes after Roux-en-Y gastric bypass compared with matched unexposed individuals, excluding those who later underwent surgery and their matched cases. 14](#_Toc199544138)

[Table S9. Outcomes after Sleeve gastrectomy compared with matched unexposed individuals, excluding those who later underwent surgery and their matched cases. 15](#_Toc199544139)

[Table S10. Number of procedures for Roux-en-Y Gastric bypass and Sleeve gastrectomy during the study period (2007-2020). 16](#_Toc199544140)

[Table S11. Body mass index and glycated hemoglobin levels at 3 and 7 years after Roux-en-Y Gastric bypass and Sleeve Gastrectomy compared to unexposed individuals. 17](#_Toc199544141)

[Supplementary Figures 18](#_Toc199544142)

[Supplementary Figure S1 Flowchart of the study cohort 18](#_Toc199544143)

[Supplementary Figure S2. Cumulative hazard curve with numbers at risk for all-cause mortality after RYGB compared to matched unexposed individuals. 19](#_Toc199544144)

[Supplementary Figure S3. Cumulative hazard curve with numbers at risk for all-cause mortality after SG compared to unexposed individuals. 20](#_Toc199544145)

[References 21](#_Toc199544146)

# **Methods**

## **Data sources**

NDR and SOReg were cross-matched on an individual level by unique personal identification numbers. Clinical characteristics (sex, age, BMI, smoking status, physical activity, diabetes duration, blood pressure and biochemical values) and type of surgery (Roux-en-Y gastric bypass or Sleeve gastrectomy) were obtained from SOReg and NDR. Socioeconomic data on nationality, educational level, civil status and income were obtained from the Longitudinal Integration Database for Health Insurance and Labour Market Studies (LISA) maintained by the government agency Statistics Sweden.^1^ Comorbidities, pharmacotherapy, and cause of death were retrieved from the National Patient Registry (NPR), the Cancer Registry, the National Prescribed Drug Registry, and the Cause of Death Registry. These registries have previously been validated and are maintained by the Swedish Board of Health and Welfare. ^2,3^ The NPR includes information on in-patient care since 1987 and outpatient doctor visits, including outpatient surgery and psychiatric care from public and private healthcare providers since 2001.

# **Supplementary tables**

## **Table S1. ICD–codes used to define baseline comorbidities up to 10 years before the index date and outcomes during follow–up 2007–2021.**

| Table S1. ICD–codes used to define baseline comorbidities up to 10 years before the index date and outcomes during follow–up 2007–2021. | | |
| --- | --- | --- |
| **Variables** | **ICD–10** | **Variable origin** |
| All–cause mortality | Everyone in the Cause of Death Register | Cause of death registry |
| Fatal cardiovascular disease | I20–I25 I61–I66, I70, I71 and entered in the Cause of Death Register | Swedish In– and Outpatient registry & Cause of Death Registry |
| Cardiovascular disease | I20–I25 I61–I66, I70, I71 | Swedish In– and Outpatient Registry |
| Acute myocardial infarction | I21 | Swedish In– and Outpatient Registry |
| Coronary heart disease | I20–I25 | Swedish In– and Outpatient Registry |
| Stroke | I61–I66 | Swedish In– and Outpatient Registry |
| Heart failure | I50 | Swedish In– and Outpatient Registry |
| Cardiac arrest | I46 | Swedish In– and Outpatient registry |
| Atrial fibrillation/flutter | I48 | Swedish In– and Outpatient Registry |
| Hypertension | I10, I11, I12, I13 | Swedish In– and Outpatient Registry |
| Venous thromboembolism | I26, I80, I81 | Swedish In– and Outpatient registry |
| Valvular heart disease | I34–I37 | Swedish In– and Outpatient registry |
| Other dysrhythmias | I44, I45, I47–I49 | Swedish In– and Outpatient registry |
| Cardiomyopathies | I420, I422, I429, I431, I432, I438, | Swedish In– and Outpatient registry |
| Diabetic microvascular complications | E112–E118, E132–138, E142–148 | Swedish In– and Outpatient registry |
| Hyperglycaemia | E110A–B, E110X, E111A–B, E111X  E120–121, E130–131, E140–141, R739 | Swedish In– and Outpatient registry |
| Hypoglycaemia (with or without coma) | E110C, E110X, E116A  E120, E130, E140, E159, E160, E161W, E162 | Swedish In– and Outpatient registry |
| Depression | F30–39 | Swedish In– and Outpatient registry |
| Anxiety disorders | F40–48 | Swedish In– and Outpatient registry |
| Alcohol use disorder | F10 | Swedish In– and Outpatient Registry |
| Other drug use disorders | F11–16, F18–19 | Swedish In– and Outpatient Registry |
| Dementia | F01–F03 | Swedish In– and Outpatient registry |
| Psychosis | F20–29 | Swedish In– and Outpatient registry |
| Behavioural disorders | F50–F52, F55, F60, F63, F68, F69, F90, F95 | Swedish In– and Outpatient registry |
| Suicide attempts | X60–84, Y10–34 | Swedish In– and Outpatient registry |
| Chronic kidney disease | N17, N18, N19, N990 | Swedish In– and Outpatient Registry |
| Transplantation | Z940–Z944, Z948, Z949 | Swedish In– and Outpatient Registry |
| Cancer | C00–97 | Swedish In– and Outpatient Registry, Cancer registry |
| All hematological diseases | D50–89 | Swedish In– and Outpatient registry |
| Anaemia^1^ | D50, D51–53, D60–64 | Swedish In– and Outpatient registry |
| Neurological disorders | G00–99 | Swedish In– and Outpatient registry |
| Musculoskeletal disorders | M00–99 | Swedish In– and Outpatient registry |
| Pulmonary diseases | J40–J46 | Swedish In– and Outpatient registry |
| Malabsorption and micronutrient deficiency | E40–44, E50–E64, E90, E83, G633, G634, K90, K911, K912, M813, M832 | Swedish In– and Outpatient registry |
| Osteoporosis | M80, M81 | Swedish In– and Outpatient registry |
| Fractures | S32–33, S42–43, S52–53, S62, S72–73 | Swedish In– and Outpatient registry |
| Other endocrine diseases | E16, E20, E21, E282, E29, E662, E87 | Swedish In– and Outpatient registry |
| Gastrointestinal reflux and ulcer | K21, K221–223, K227, K25–28 | Swedish In– and Outpatient registry |
| Hernia (with or without strangulation) | K40–46 | Swedish In– and Outpatient registry |
| Functional gastrointestinal disorders | K58–59, K30 | Swedish In– and Outpatient registry |
| Liver diseases | K760, K72–K74 | Swedish In– and Outpatient registry |
| Gallbladder and pancreatic diseases | K80–85, K860, K861, K868, K869 | Swedish In– and Outpatient registry |
| Lower gastrointestinal disorders | K50–52, | Swedish In– and Outpatient registry |
| Abdominal pain | R10 | Swedish In– and Outpatient registry |
| Skin diseases | L20–L30, L40, L50–L54, L70–L73, L89 | Swedish In– and Outpatient registry |
|  |  |  |
| Bowel obstruction | K56, K913 | Swedish In– and Outpatient registry |
| Gastrointestinal leakage | K631, K65, K918 | Swedish In– and Outpatient registry |
| Surgical wound complications | K430, K439, T810, T813, T814, T818, T819 | Swedish In– and Outpatient registry |
| ^1^Separated in follow–up analyses. | | |

| Table S2. ATC–codes used to define baseline prescription therapies. | | |
| --- | --- | --- |
| **Prescription drugs** | **ATC code** | **Variable origin** |
| Gastrointestinal medication | A02AD01, A02B, A03AX13, A04, A06A, A07 | Prescription drug registry |
| Weight–loss agents | A08 | Prescription drug registry |
| Antihyperglycemic agents | A10 | Prescription drug registry |
| Other vitamin supplements | A11C, A11D, A11E | Prescription drug registry |
| Calcium and/or vitamin D supplements | A12 | Prescription drug registry |
| Anticoagulant agents | B01A | Prescription drug registry |
| Anaemia medication | B03 | Prescription drug registry |
| Antihypertensive agents | C02, C03, C07, C08, C09 | Prescription drug registry |
| Antiarrhythmia agents | C01B | Prescription drug registry |
| Other cardiovascular agents | C01AA, C01C, C01DX22 | Prescription drug registry |
| Vasodilator agents | C01DA | Prescription drug registry |
| Hypolipidemic agents | C10 | Prescription drug registry |
| Corticosteroids | H02AB | Prescription drug registry |
| Other endocrine agents | H03AA01, H03AA02, H05A, H05B | Prescription drug registry |
| Antineoplastic agents | L01–L04 | Prescription drug registry |
| Musculoskeletal agents | M01, M02, M04, M05B | Prescription drug registry |
| Analgesic agents | N02, M01A | Prescription drug registry |
| Anticonvulsive agents | N03 | Prescription drug registry |
| Neuroleptic agents | N05A | Prescription drug registry |
| Sedatives and hypnotics | N05B, N05C | Prescription drug registry |
| Antidepressive agents | N06A | Prescription drug registry |
| Central stimulant | N06BA | Prescription drug registry |
| Cognitive enhancers | N06D | Prescription drug registry |
| Inhalation and respiratory therapies | R03 | Prescription drug registry |

## **Table S2. ATC–codes used to define baseline prescription therapies.**

## **Table S3. Continuation of Table 1: baseline comorbidities and prescription drugs.**

| **Table S3. Continuation of Table 1: baseline comorbidities and prescription drugs.** | | | | | | |
| --- | --- | --- | --- | --- | --- | --- |
|  | **RYGB^1^**  **(n=7294)** | **Unexposed individuals**  **(n=7294)** | **SMD^3^** | **SG^2^**  **(n=1105)** | **Unexposed individuals**  **(n=1105)** | **SMD^3^** |
| **Other diagnoses** |  |  |  |  |  |  |
| Transplantation n (%) | 13 (0·2) | 22 (0·3) | **0·025** | 7 (0·6) | 3 (0·3) | **0·054** |
| Valvular heart disease n (%) | 59 (0·8) | 64 (0·9) | **0·007** | 10 (0·9) | 10 (0·9) | **<0·001** |
| Other dysrhythmias n (%) | 418 (5·7) | 389 (5·3) | **0·017** | 59 (5·3) | 55 (5·0) | **0·016** |
| Cardiomyopathy n (%) | 49 (0·7) | 86 (1·2) | **0·053** | 10 (0·9) | 7 (0·6) | **0·031** |
| Functional gastrointestinal disorders n (%) | 476 (6·5) | 433 (5·9) | **0·024** | 97 (8·8) | 79 (7·1) | **0·060** |
| Haematological diseases n (%) | 167 (2·3) | 204 (2·8) | **0·032** | 34 (3·1) | 28 (2·5) | **0·033** |
| Neurological disorders n (%) | 2704 (37·1) | 1752 (24·0) | **0·286** | 390 (35·3) | 262 (23·7) | **0·256** |
| Musculoskeletal disorders n (%) | 3464 (47·5) | 2784 (38·2) | **0·189** | 607 (54·9) | 449 (40·6) | **0·289** |
| Dementia n (%) | 2 (0·0) | 11 (0·2) | **0·041** | 0 (0·0) | 0 (0·0) | **<0·001** |
| Behavioural disorders n (%) | 319 (4·4) | 396 (5·4) | **0·049** | 66 (6·0) | 78 (7·1) | **0·044** |
| Psychosis n (%) | 73 (1·0) | 317 (4·3) | **0·209** | 15 (1·4) | 54 (4·9) | **0·204** |
| Skin diseases n (%) | 943 (12·9) | 866 (11·9) | **0·032** | 155 (14·0) | 145 (13·1) | **0·026** |
| Endocrine disorders n (%) | 378 (5·2) | 401 (5·5) | **0·014** | 83 (7·5) | 72 (6·5) | **0·039** |
| Fracture n (%) | 485 (6·6) | 440 (6·0) | **0·025** | 66 (6·0) | 73 (6·6) | **0·026** |
| Surgical complications wound n (%) | 127 (1·7) | 138 (1·9) | **0·011** | 31 (2·8) | 23 (2·1) | **0·047** |
| Lower gastrointestinal disorders n (%) | 154 (2·1) | 181 (2·5) | **0·025** | 53 (4·8) | 24 (2·2) | **0·143** |
| **Prescription therapies** |  |  |  |  |  |  |
| Other vitamin supplements n (%) | 416 (5·7) | 313 (4·3) | **0·065** | 84 (7·6) | 80 (7·2) | **0·01** |
| Antiarrhythmia agents n (%) | 28 (0·4) | 23 (0·3) | **0·012** | 2 (0·2) | 3 (0·3) | **0·019** |
| Other cardiovascular agents (%) | 89 (1·2) | 118 (1·6) | **0·034** | 15 (1·4) | 13 (1·2) | **0·016** |
| Vasodilator agents n (%) | 207 (2·8) | 294 (4·0) | **0·066** | 21 (1·9) | 36 (3·3) | **0·086** |
| Corticosteroids n (%) | 443 (6·1) | 534 (7·3) | **0·050** | 83 (7·5) | 77 (7·0) | **0·021** |
| Other endocrine agents n (%) | 841 (11·5) | 860 (11·8) | **0·008** | 152 (13·8) | 119 (10·8) | **0·091** |
| Antineoplastic agents n (%) | 170 (2·3) | 225 (3·1) | **0·046** | 36 (3·3) | 32 (2·9) | **0·021** |
| Musculoskeletal agents n (%) | 2581 (35·4) | 2225 (30·5) | **0·104** | 401 (36·3) | 270 (24·4) | **0·260** |
| Analgesic agents n (%) | 5350 (73·3) | 2895 (39·7) | **0·722** | 865 (78·3) | 385 (34·8) | **0·975** |
| Anticonvulsive agents n (%) | 420 (5·8) | 456 (6·3) | **0·021** | 98 (8·9) | 80 (7·2) | **0·060** |
| Neuroleptic agents n (%) | 241 (3·3) | 504 (6·9) | **0·164** | 43 (3·9) | 96 (8·7) | **0·199** |
| Central stimulant n (%) | 74 (1·0) | 75 (1·0) | **0·001** | 21 (1·9) | 16 (1·4) | **0·035** |
| Cognitive enhancers n (%) | 3 (0·0) | 5 (0·1) | **0·012** | 0 (0·0) | 1 (0·1) | **0·043** |
| Inhalation and respiratory therapies n (%) | 913 (12·5) | 1246 (17·1) | **0·129** | 164 (14·8) | 200 (18·1) | **0·088** |
| ^1^RYGB = Roux–en–Y Gastric bypass· ^2^SG = Sleeve gastrectomy· ^3^SMD = Standardised mean difference· ^4^Concentrations of glycated hemoglobin are based on values from the International Federation of Clinical Chemistry· | | | | | | |

| **Table S4. Continuation of Table 2: Incidence rates and adjusted Hazard ratios for each outcome after Roux–en–Y Gastric Bypass compared with matched unexposed individuals.** | | | | |
| --- | --- | --- | --- | --- |
|  | **Unexposed individuals**  **(n=7294)** | **RYGB^1^**  **(n=7294)** | **aHR [95% CI]^2^** | **p–value** |
| Other diagnoses |  |  |  |  |
| Cardiomyopathy | 17·6 [14·4–21·3]^3^ | 12·4 [9·7–15·5] | 0·88 [0·63–1·22] | 0·426 |
| Cardiac arrest | 18·8 [15·5–22·7] | 9·6 [7·3–12·5] | 0·49 [0·34–0·70] | <0·001 |
| Neurological disorders | 408·3 [390·5–426·7] | 367·9 [351·3–385·1] | 0·78 [0·72–0·83] | <0·001 |
| Musculoskeletal disorders | 760·0 [733·6–787·1] | 920·5 [890·8–950·8] | 1·11 [1·05–1·17] | <0·001 |
| Psychosis | 51·9 [46·2–58·2] | 12·5 [9·9–15·7] | 0·60 [0·44–0·81] | 0·001 |
| Behavioural disorders | 65·5 [59·1–72·5] | 71·0 [64·3–78·2] | 1·49 [1·26–1·75] | <0·001 |
| Dementia | 7·8 [5·7–10·4] | 4·0 [2·6–5·9] | 0·53 [0·29–0·96] | 0·037 |
| Endocrine disorders | 102·3 [94·1–111·0] | 100·7 [92·7–109·3] | 0·96 [0·84–1·09] | 0·502 |
| Lower gastrointestinal disorders | 30·9 [26·5–35·7] | 31·2 [26·9–36·1] | 0·91 [0·72–1·13] | 0·380 |
| Other liver diseases | 32·4 [28·0–37·4] | 22·9 [19·2–27·1] | 0·63 [0·49–0·81] | <0·001 |
| All haematological diseases | 55·5 [49·6–61·9] | 87·9 [80·4–95·8] | 1·62 [1·39–1·90] | <0·001 |
| Skin diseases | 175·9 [164·9–187·5] | 152·5 [142·4–163·1] | 0·84 [0·76– 0·94] | 0·001 |
| ^1^RYGB = Roux–en–Y Gastric Bypass. ^2^aHR = adjusted Hazard Ratio, CI = Confidence interval, between RYGB and matched unexposed individuals. ^3^Incidence rates per 10,000 person-years with confidence intervals. | | | | |

## **Table S4. Continuation of Table 2: Incidence rates and adjusted Hazard ratios for each outcome after Roux–en–Y Gastric Bypass compared with matched unexposed individuals.**

## **Table S5.** **Continuation of Table 3: Incidence rates and adjusted Hazard ratios for each outcome after Sleeve Gastrectomy compared with matched unexposed individuals.**

| **Table S5.** **Continuation of Table 3: Incidence rates and adjusted Hazard ratios for each outcome after Sleeve Gastrectomy compared with matched unexposed individuals.** | | | | |
| --- | --- | --- | --- | --- |
|  | **Unexposed individuals**  **(n=1105)** | **SG^1^**  **(n=1105)** | **aHR [95% CI]^2^** | **p–value** |
| Other diagnoses |  |  |  |  |
| Cardiomyopathy | 20·6 [9·9–37·9]^3^ | 20·4 [9·8–37·6] | 0·57 [0·19–1·72] | 0·320 |
| Cardiac arrest | 14·4 [5·8–29·6] | 4·1 [0·5–14·7] | 0·40 [0·04–3·89] | 0·432 |
| Neurological disorders | 427·3 [367·8–493·7] | 402·6 [345·5–466·5] | 0·78 [0·61–0·99] | 0·043 |
| Musculoskeletal disorders | 733·8 [651·8–823·3] | 1207·2 [1095·6–1327·0] | 1·41 [1·18–1·68] | <0·001 |
| Psychosis | 104·4 [77·3–138·1] | 28·8 [15·8–48·4] | 0·90 [0·46–1·76] | 0·755 |
| Behavioural disorders | 112·7 [84·5–147·5] | 102·6 [75·9–135·7] | 1·08 [0·63–1·87] | 0·760 |
| Dementia | 0·0 [0·0–7·6] | 0·0 [0·0–7·5] | – | – |
| Endocrine disorders | 120·3 [91·1–155·9] | 104·5 [77·6–137·8] | 0·83 [0·53–1·30] | 0·407 |
| Lower gastrointestinal disorders | 33·2 [19·0–53·9] | 86·6 [62·2–117·5] | 1·46 [0·78–2·70] | 0·235 |
| Other liver diseases | 54·1 [35·4–79·30] | 36·9 [21·9–58·35] | 0·48 [0·23–0·97] | 0·041 |
| All haematological diseases | 58·6 [38·9–84·64] | 53·5 [35·0–78·41] | 1·11 [0·60–2·05] | 0·742 |
| Skin diseases | 159·9 [125·5–200·7] | 232·2 [190·1–280·9] | 1·75 [1·24–2·48] | 0·002 |
| ^1^SG = Sleeve gastrectomy. ^2^aHR = adjusted Hazard Ratio, CI Confidence = interval, between SG and matched unexposed individuals. ^3^Incidence rates per 10,000 person-years with confidence intervals. | | | | |

## **Table S6.** **Number of events and person–years for each outcome after Roux–en–Y Gastric Bypass compared with matched unexposed individuals.**

| **Table S6.** **Number of events and person–years for each outcome after Roux–en–Y Gastric Bypass compared with matched unexposed individuals.** | | |
| --- | --- | --- |
|  | **Unexposed individuals**  **(n=7294)** | **RYGB^1^**  **(n=7294)** |
| All–cause mortality | 703 (59025) | 475 (60283) |
| Cardiovascular disease |  |  |
| Cardiovascular disease | 1051 (54067) | 947 (55203) |
| Fatal cardiovascular disease | 251 (59025) | 137 (60283) |
| Acute myocardial infarction | 273 (57954) | 166 (59589) |
| Coronary heart disease | 766 (55196) | 711 (56182) |
| Heart failure | 684 (56362) | 384 (58742) |
| Stroke | 283 (57908) | 236 (59357) |
| Atrial fibrillation | 617 (56276) | 549 (57597) |
| Other dysrhythmias | 786 (55405) | 702 (56952) |
| Valvular heart disease | 150 (58339) | 138 (59749) |
| Hypertension | 3303 (40471) | 2814 (43782) |
| Venous thromboembolism | 304 (57768) | 228 (59331) |
| Diabetes–related disorders |  |  |
| Hypoglycaemia | 52 (58975) | 52 (60232) |
| Hyperglycaemia | 2251 (50745) | 1585 (53862) |
| Diabetic microvascular complications | 2112 (51415) | 1523 (53987) |
| Psychiatric disorders |  |  |
| Depression | 714 (54705) | 897 (54850) |
| Anxiety disorders | 848 (54293) | 1012 (54813) |
| Alcohol use disorders | 207 (57951) | 536 (57781) |
| Other drug use disorders | 149 (58278) | 248 (59006) |
| Suicide attempt | 0 (59025) | 31 (60283) |
| Other diseases |  |  |
| Chronic kidney disease | 643 (56674) | 377 (58834) |
| Malabsorption and micronutrient deficiency | 414 (57286) | 838 (55961) |
| Anaemia | 701 (56166) | 1049 (55524) |
| Cancer | 697 (56339) | 575 (57899) |
| Pulmonary disease | 845 (54623) | 541 (57401) |
| Osteoporosis | 67 (58770) | 102 (59943) |
| Fractures | 550 (56785) | 984 (56132) |
| Gastrointestinal diseases |  |  |
| Hernia | 524 (56201) | 811 (55602) |
| Gastrointestinal reflux and ulcer | 364 (58293) | 631 (58556) |
| Abdominal pain | 1519 (54091) | 2536 (49953) |
| Non–alcoholic Fatty Liver Disease | 95 (58602) | 57 (60079) |
| Gallbladder and pancreatic diseases | 457 (56852) | 822 (56167) |
| Surgical complications |  |  |
| Bowel obstruction | 105 (58898) | 406 (59562) |
| Gastrointestinal leakage | 57 (58970) | 125 (60038) |
| Surgical wound complications | 177 (58651) | 319 (59450) |
| Other diagnoses |  |  |
| Cardiomyopathy | 103 (58605) | 74 (59927) |
| Cardiac arrest | 111 (58947) | 58 (60219) |
| Neurological disorders | 1978 (48442) | 1848 (50231) |
| Musculoskeletal disorders | 3132 (41210) | 3647 (39621) |
| Psychosis | 297 (57134) | 75 (59836) |
| Behavioural disorders | 373 (56911) | 412 (58001) |
| Dementia | 46 (58932) | 24 (60214) |
| Endocrine disorders | 581 (56799) | 581 (57689) |
| Lower gastrointestinal disorders | 179 (58011) | 185 (59260) |
| Other liver diseases | 189 (58324) | 137 (59825) |
| All haematological diseases | 320 (57678) | 510 (58046) |
| Skin diseases | 952 (54113) | 848 (55619) |
| ^1^RYGB = Roux–en–Y Gastric Bypass. ^2^Number of events and (person–years). | | |

## **Table S7.** **Number of events and person–years for each outcome after Sleeve Gastrectomy compared with matched unexposed individuals.**

| **Table S7.** **Number of events and person–years for each outcome after Sleeve Gastrectomy compared with matched unexposed individuals.** | | |
| --- | --- | --- |
|  | **Unexposed individuals**  **(n=1105)** | **SG^1^**  **(n=1105)** |
| All–cause mortality | 38 (4877)^2^ | 31 (4922) |
| Cardiovascular disease |  |  |
| Cardiovascular disease | 86 (4627) | 89 (4655) |
| Fatal cardiovascular disease | 13 (4877) | 6 (4922) |
| Acute myocardial infarction | 18 (4835) | 14 (4893) |
| Coronary heart disease | 64 (4681) | 70 (4712) |
| Heart failure | 47 (4779) | 36 (4826) |
| Stroke | 23 (4825) | 16 (4887) |
| Atrial fibrillation | 63 (4704) | 48 (4781) |
| Other dysrhythmias | 73 (4688) | 54 (4770) |
| Valvular heart disease | 10 (4847) | 10 (4884) |
| Hypertension | 314 (3900) | 272 (4054) |
| Venous thromboembolism | 28 (4814) | 24 (4842) |
| Diabetes–related disorders |  |  |
| Hypoglycaemia | 4 (4872) | 3 (4917) |
| Hyperglycaemia | 220 (4389) | 148 (4523) |
| Diabetic microvascular complications | 203 (4418) | 143 (4536) |
| Psychiatric disorders |  |  |
| Depression | 77 (4614) | 77 (4632) |
| Anxiety disorders | 102 (4544) | 105 (4578) |
| Alcohol use disorders | 24 (4818) | 22 (4846) |
| Other drug use disorders | 10 (4849) | 21 (4864) |
| Suicide attempt | 0 (4877) | 1 (4922) |
| Other diseases |  |  |
| Chronic kidney disease | 54 (4745) | 57 (4740) |
| Malabsorption and micronutrient deficiency | 26 (4803) | 47 (4764) |
| Anaemia | 43 (4744) | 46 (4810) |
| Cancer | 65 (4703) | 59 (4795) |
| Pulmonary disease | 76 (4662) | 41 (4792) |
| Osteoporosis | 5 (4869) | 4 (4912) |
| Fractures | 39 (4760) | 58 (4759) |
| Gastrointestinal diseases |  |  |
| Hernia | 49 (4765) | 74 (4671) |
| Gastrointestinal reflux and ulcer | 25 (4851) | 45 (4869) |
| Abdominal pain | 147 (4512) | 211 (4321) |
| Non–alcoholic Fatty Liver Disease | 15 (4843) | 10 (4898) |
| Gallbladder and pancreatic diseases | 42 (4742) | 65 (4751) |
| Surgical complications |  |  |
| Bowel obstruction | 13 (4876) | 17 (4916) |
| Gastrointestinal leakage | 5 (4877) | 9 (4916) |
| Surgical wound complications | 14 (4867) | 26 (4853) |
| Other diagnoses |  |  |
| Cardiomyopathy | 10 (4852) | 10 (4892) |
| Cardiac arrest | 7 (4877) | 2 (4922) |
| Neurological disorders | 184 (4306) | 177 (4397) |
| Musculoskeletal disorders | 290 (3952) | 429 (3554) |
| Psychosis | 49 (4692) | 14 (4858) |
| Behavioural disorders | 53 (4701) | 49 (4774) |
| Dementia | 0 (4877) | 0 (4922) |
| Endocrine disorders | 57 (4738) | 50 (4785) |
| Lower gastrointestinal disorders | 16 (4823) | 41 (4733) |
| Other liver diseases | 26 (4808) | 18 (4876) |
| All haematological diseases | 28 (4781) | 26 (4858) |
| Skin diseases | 74 (4629) | 106 (4565) |
| ^1^SG = Sleeve gastrectomy. ^2^Number of events and (person–years). | | |

## **Table S8. Outcomes after Roux-en-Y gastric bypass compared with matched unexposed individuals, excluding those who later underwent surgery and their matched cases.**

| **Table S8. Outcomes after Roux-en-Y gastric bypass compared with matched unexposed individuals, excluding those who later underwent surgery and their matched cases.** | | | | |
| --- | --- | --- | --- | --- |
|  | **RYGB^1^**  **(n=6598)** | **Unexposed individuals**  **[n=6504)** | **aHR [95% CI]^2^** | **p-value** |
| All-cause mortality | 79·4 [72·2–87·2] ^3^ | 137·1 [127·1–147·6] | 0·55 [0·48–0·63] | <0·001 |
| Cardiovascular diseases |  |  |  |  |
| Fatal cardiovascular disease | 23·8 [19·2–28·3] | 48·0 [42·2–54·4] | 0·48 [0·38–0·61] | <0·001 |
| Cardiovascular disease | 175·0 [163·6–187·0] | 204·6 [191·8–217·9] | 0·88 [0·80–0·98] | 0·015 |
| Heart failure | 66·8 [60·1–74·1] | 133·9 [123·8–144·6] | 0·47 [0·41–0·54] | <0·001 |
| Diabetes-related disorders |  |  |  |  |
| Hyperglycaemia | 293·1 [278·2–308·7] | 460·3 [440·4–480·7] | 0·56 [0·52–0·60] | <0·001 |
| Diabetic microvascular complications | 280·6 [266·0–295·8] | 425·6 [406·7–445·2] | 0·57 [0·53–0·61] | <0·001 |
| Psychiatric disorders |  |  |  |  |
| Depression | 164·8 [153·7–176·5] | 127·5 [117·6–138·1] | 1·37 [1·21–1·54] | <0·001 |
| Alcohol use disorders | 95·1 [87·0–103·8] | 33·5 [28·7–39·0] | 3·14 [2·60–3·80] | <0·001 |
| Other diseases |  |  |  |  |
| Chronic kidney disease | 64·6 [58·0–71·8] | 122·9 [113·3–133·1] | 0·49 [0·42–0·57] | <0·001 |
| Malabsorption and micronutrient deficiency | 152·4 [141·9–163·5] | 67·3 [60·3–74·9] | 2·03 [1·91–2·54] | <0·001 |
| Gastrointestinal diseases |  |  |  |  |
| Gastrointestinal reflux and ulcer | 107·1 [98·5–116·2] | 55·2 [48·9–62·0] | 1·94 [1·66–2·56] | <0·001 |
| Abdominal pain | 503·2 [482·8–524·2] | 258·4 [244·1–273·3] | 2·08 [1·92–2·25] | <0·001 |
| Surgical complications |  |  |  |  |
| Bowel obstruction | 69·4 [62·5–76·7] | 14·7 [11·5–18·7] | 4·45 [3·41–5·0] | <0·001 |
| ^1^RYGB = Roux-en-Y Gastric Bypass. ^2^ aHR = adjusted Hazard Ratio, CI = Confidence interval, between RYGB and unexposed individuals. ^3^Incidence rates per 10·000 person-years with confidence intervals. | | | | |

## **Table S9.** **Outcomes after Sleeve gastrectomy compared with matched unexposed individuals, excluding those who later underwent surgery and their matched cases.**

| **Table S9. Outcomes after Sleeve gastrectomy compared with matched unexposed individuals, excluding those who later underwent surgery and their matched cases.** | | | | |
| --- | --- | --- | --- | --- |
|  | **SG^1^**  **(n=966)** | **Unexposed individuals**  **[n=1060)** | **aHR [95% CI]^2^** | **p-value** |
| All-cause mortality | 63·2 [41·6–91·9] ^3^ | 82·2 [58·2–112·8] | 1·01 [0·56–1·84] | 0·965 |
| Cardiovascular diseases |  |  |  |  |
| Fatal cardiovascular disease | 12·2 [4·5–26·5] | 26·7 [14·2–45·6] | 0·61 [0·18–2·07] | 0·430 |
| Cardiovascular disease | 191·2 [153·5–235·3] | 185·9 [148·7–229·6] | 1·35 [0·94–1·94] | 0·100 |
| Heart failure | 74·6 [52·2–103·3] | 98·4 [72·3–130·8] | 0·85 [0·52–1·39] | 0·520 |
| Diabetes-related disorders |  |  |  |  |
| Hyperglycaemia | 327·2 [276·6–384·4] | 501·3 [437·2–572·1] | 0·56 [0·52–0·60] | <0·001 |
| Diabetic microvascular complications | 315·3 [265·7–371·4] | 459·5 [394·4–527·2] | 0·57 [0·53–0·61] | <0·001 |
| Psychiatric disorders |  |  |  |  |
| Depression | 166·2 [131·2–207·9] | 166·9 [131·7–207·8] | 0·97 [0·63–1·48] | 0·880 |
| Alcohol use disorders | 45·4 [28·5–68·7] | 49·8 [31·9–74·1] | 0·79 [0·38–1·65] | 0·533 |
| Other diseases |  |  |  |  |
| Chronic kidney disease | 120·2 [91·1–155·8] | 113·8 [85·5–148·5] | 1·15 [0·72–1·84] | 0·553 |
| Malabsorption and micronutrient deficiency | 98·7 [72·5–131·2] | 54·1 [35·4–79·3] | 2·53 [1·39–4·60] | 0·002 |
| Gastrointestinal diseases |  |  |  |  |
| Gastrointestinal reflux and ulcer | 92·4 [67·4–123·7] | 51·5 [33·3–76·1] | 1·09 [0·60–1·99] | 0·771 |
| Abdominal pain | 488·3 [424·6–558·8] | 325·8 [275·3–383·0] | 1·64 [1·28–2·18] | 0·001 |
| Surgical complications |  |  |  |  |
| Bowel obstruction | 34·6 [20·1–55·4] | 26·6 [14·2–45·6] | 0·97 [0·37–2·57] | 0·951 |
| ^1^SG = Sleeve gastrectomy. ^2^ aHR = adjusted Hazard Ratio, CI = Confidence interval, between SG and unexposed individuals. ^3^Incidence rates per 10·000 person-years with confidence intervals. | | | | |

## **Table S10.** **Number of procedures for Roux-en-Y Gastric bypass and Sleeve gastrectomy during the study period (2007-2020).**

| **Table S10. Number of procedures of Roux-en-Y Gastric bypass and Sleeve gastrectomy during the study period (2007-2020).** | | |
| --- | --- | --- |
|  | Roux-en-Y Gastric bypass  (n=7294) | Sleeve gastrectomy  (n=1105) |
| Year |  |  |
| 2007 | 71 (100%) | 0 (100%) |
| 2008 | 287 (99·3 %) | 2 (0·7%) |
| 2009 | 456 (100%) | 0 (100%) |
| 2010 | 894 (99·7%) | 3 (0·3%) |
| 2011 | 1134 (99·1%) | 10 (0·9%) |
| 2012 | 880 (97·5%) | 23 (2·5%) |
| 2013 | 729 (96·2%) | 29 (3·8%) |
| 2014 | 666 (89·8%) | 76 (10·2%) |
| 2015 | 551 (79·5%) | 142 (20·5%) |
| 2016 | 439 (76·5%) | 135 (23·5%) |
| 2017 | 359 (67·6%) | 172 (32·4%) |
| 2018 | 333 (61·8%) | 206 (38·2%) |
| 2019 | 280 (62·2%) | 170 (37·8%) |
| 2020 | 215 (61·1%) | 137 (38·9%) |

## **Table S11.** **Body mass index and glycated hemoglobin levels at 3 and 7 years after Roux-en-Y Gastric bypass and Sleeve Gastrectomy compared to unexposed individuals.**

| **Table S11.** **Body mass index and glycated haemoglobin levels at 3 and 7 years after Roux-en-Y Gastric bypass and Sleeve Gastrectomy compared to unexposed individuals.** | | | | |
| --- | --- | --- | --- | --- |
| **3 years** | **RYGB^1^**  **(n=3761)** | **Unexposed individuals**  **(n=5172)** | **SG^2^**  **(n=494)** | **Unexposed individuals**  **(n=546)** |
| Body mass index (mean (SD)) | 31·3 (5·7) | 38·8 (6·5) | 33·8 (5·8) | 38·3 (6·4) |
| % Total weight loss (mean (SD)) | 22·8 (10·9) | 4·5 (10·8) | 15·3 (10·6) | 3·7 (4·0) |
| Glycated haemoglobin levels (mean (SD)) | 47 (13) | 57 (16) | 48 (14) | 56 (16) |
| **7 years** | (n=2651) | (n=3457) | (n=117) | (n=84) |
| Body mass index (mean (SD)) | 32·2 (5·7) | 38·1 (6·3) | 35·0 (5·7) | 37·5 (6·5) |
| % Total weight loss^4^ (mean (SD)) | 20·8 (10·7) | 6·2 (11·6) | 13·4 (10·1) | 4·5 (10·5) |
| Glycated haemoglobin^3^ levels (mean (SD)) | 51 (13) | 59 (16) | 54 (14) | 60 (16) |
| ^1^RYGB = Roux–en–Y Gastric bypass. ^2^SG = Sleeve gastrectomy. ^3^ Glycated haemoglobin = HbA1c. ^4^ % Total weight loss = 100*(baseline weight – current weight / baseline weight). | | | | |

# **Supplementary Figures**

## **Supplementary Figure S1 Flowchart of the study cohort**


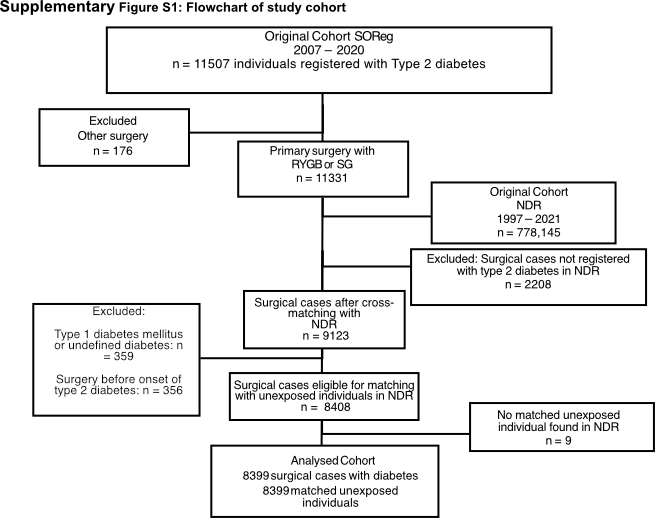


## **
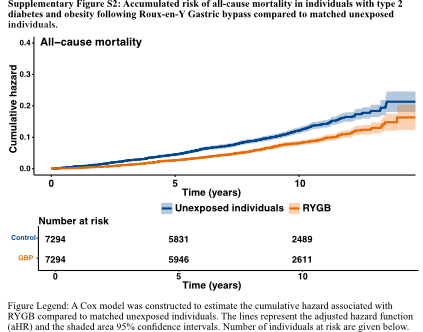
Supplementary Figure S2. Cumulative hazard curve with numbers at risk for all-cause mortality after RYGB compared to matched unexposed individuals.**

## **Supplementary Figure S3. Cumulative hazard curve with numbers at risk for all-cause mortality after SG compared to unexposed individuals.**

**
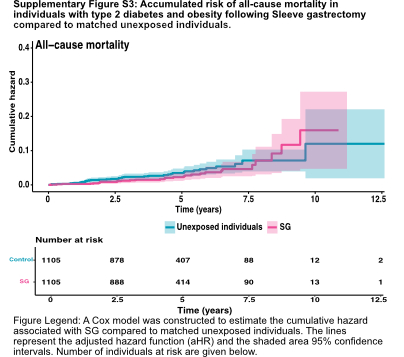
**

##

# **References**

1. Ludvigsson JF, Svedberg P, Olén O, Bruze G, Neovius M. The longitudinal integrated database for health insurance and labour market studies (LISA) and its use in medical research. Eur J Epidemiol. 2019;34(4):423-37.

2. Ludvigsson JF, Andersson E, Ekbom A, Feychting M, Kim J-L, Reuterwall C, et al. External review and validation of the Swedish national inpatient register. BMC Public Health. 2011;11(1):450.

3. Wettermark B, Hammar N, Michaelfored C, Leimanis A, Otterblad Olausson P, Bergman U, et al. The new Swedish Prescribed Drug Register—Opportunities for pharmacoepidemiological research and experience from the first six months. Pharmacoepidemiol Drug Saf. 2007;16(7):726-35.
